# Vitamin E Acetate as a Plausible Cause of Acute Vaping-related Illness

**DOI:** 10.7759/cureus.6350

**Published:** 2019-12-11

**Authors:** F Brian Boudi, Sonia Patel, Ava Boudi, Connie Chan

**Affiliations:** 1 Cardiology, University of Arizona College of Medicine, Phoenix, USA; 2 Psychology, Arizona State University, Phoenix, USA; 3 Miscellaneous, Arizona State University, Phoenix, USA; 4 Internal Medicine, University of Arizona College of Medicine, Phoenix, USA

**Keywords:** evali, vaping, lung injury, vitamin e

## Abstract

The United States Federal Centers for Disease Control and Prevention (CDC) has been working with state investigators on reported cases of lung illnesses linked to e-cigarette or vaping products. Symptoms of difficulty breathing, shortness of breath, chest pains, gastrointestinal sickness leading to serious lung damage and death has been linked to the risk behavior of using vaping products bought on the streets in healthy young people. CDC has detected vitamin E acetate as a chemical of concern among people with the lung injury. Vitamin E acetate is a condensing agent in vaping products, and all injured lung fluid samples appear to harbor this agent. The mysterious outbreak is identified in individuals vaping within the 90 days, ranging over a few days to developing over several weeks. There is growing evidence that vaping is hazardous to your health including immediate health dangers such as death from respiratory causes, long term health effects, cardiovascular events, depression which increases the risk of suicidal thoughts and suicide. This review article summarizes the growing knowledge of acute respiratory complications associated with vaping.

## Introduction and background

“Vaping” (i.e., heating) is the practice of inhaling an aerosol created by heating a liquid or wax containing substances, such as nicotine, cannabinoids such as tetrahydrocannabinol (THC), cannabidiol, additives, and flavorings such as propylene glycol, glycerol and flavored nicotine [1]. Vaping product use associated lung injury (EVALI, also called vaping associated pulmonary injury [VAPI]) is an acute or subacute respiratory illness with damage to the alveoli that can be severe and life-threatening [2-3]. There are many available devices to generate this aerosol, including battery-operated electronic cigarettes, e-cigarettes, vape pens, or vape mods [4]. While the specific cause of vaping-related illnesses has been widely debated among the general public and physicians, new research points to vitamin E acetate as a potential culprit in the vape-related lung illness outbreak. Vitamin E acetate is an oily chemical commonly added to THC vaping liquids to dilute or thicken them; the substance has been acknowledged as a potential toxin of concern by the Center for Disease Control and Prevention (CDC), due to its ability to remain in the lungs for long periods of time, and therefore cause complications in the lungs. The identification of vitamin E acetate as a harmful chemical in vaping liquid allows physicians to implement more concentrated care when treating vape-related illnesses and allows for the general public to gain a better understanding of the harmful substances in vaping products.

Electronic cigarettes were first developed in China in the early 2000s and introduced to the US market in 2007 [5]. In the US, the product experienced explosive growth, with the number of electronic cigarette users doubling every year between 2008 and 2012. While traditional cigarettes are smoked through combustion, e-cigarettes are "vaped," and the resultant aerosols potentially contain a reduced number of potentially toxic chemicals, such as nicotine and flavorings such as diacetyl and cinnamaldehyde, as well as byproducts such as formaldehyde and acrolein caused by the potential overheating of propylene glycol, and glycerin.

The Tobacco Control Act stipulates that the Food and Drug Administration shall have the authority to establish product standards for the nicotine content of combusted tobacco products. Vaping was initially marketed as a smoking cessation aid to help with cessation of cigarette smoking. E-cigarettes first took public attention in the mass media for unexpectedly blowing up, causing burns and severe facial damage [6]. In the past decade, a number of alternative vaping products have rapidly gained consumer demand, especially in, adolescents, due to the belief that they are much safer (lower nicotine content) than traditional cigarettes, choice of advertisements different flavors and ease of access to electronic nicotine delivery systems (such as e-cigarettes and vape pens). Except for menthol, the use of flavor additives has been banned from traditional cigarettes, whereas e-cigarettes are marketed in over 7,000 different flavors. Many of those flavors are found in candy and popular soft drinks and, because adolescents are familiar with such flavors, e-cigarettes are appealing to them. Tobacco smoking is associated with vascular endothelial dysfunction in a causative and dose-dependent manner [7]. Data from 5,400 smokers and 2,025 former smokers have found that the average number of cigarettes smoked per day by people who regularly used e-cigarettes fell by 4.4 over about two years, compared with only 2.7 for those who did not use e-cigarettes. Sixty-seven percent more e-cigarette users than non-users quit smoking altogether. However, there were 70% more relapses among former smokers who used e-cigarettes than among those who did not use the devices [8].

One of the most commonly known/used vape products by adolescents is the JUUL, which has twice the amount of nicotine concentrate as other e-cigarette brands, according to the American Academy of Family Physicians. The nicotine level in one JUUL pod is equivalent to that of one pack of cigarettes, and the pod consists of a mixture of nicotine salts, glycerol, propylene glycol, benzoic acid, and various flavorings [9]. Juul Labs, Inc. is an American electronic cigarette company that became the most popular e-cigarette in the United States at the end of 2017 with a market share of 72% as of September 2018 [10]. The three main reasons e-cigarettes are attractive to young people are the belief that vaping is less harmful than smoking, and they have a lower per-user cost than traditional cigarettes and flavorings (i.e., watermelon or apple pie). Although vaping has become an integral part of the adolescent culture in recent years, the medical effects of vaping have only recently been brought to light. Many adolescents, especially, have experienced lung illnesses related to vaping, and there have been a number of people that have died due to complications from vaping-related illnesses. As of the last week of October 2019, the CDC reported that there were 1,888 cases of vape-related illnesses and 37 deaths [11]. The death toll has risen since then, and U.S. health officials have confirmed that there have been at least 39 deaths that can be attributed to vaping illnesses, along with 2,051 of EVALI/VAPI cases. Clinicians have been instructed to remain suspicious of influenza and other respiratory infections in all patients who are experiencing respiratory symptoms that have a history of e-cigarette or vape use [12]. Physicians have been treating vaping-related illnesses keeping in mind that each vape-related injury may be attributed to lung injuries, infections, or both. The possible causes for these complications are still widely debated, as many believe these illnesses could be caused by a multitude of ingredients found in vape. A case study of hard-metal pneumoconiosis, published in the European Respiratory Journal, researchers tested the patient's e-cigarette, which used with cannabis, found cobalt in the vapor was released, including other toxic metals-nickel, aluminum, manganese, lead, and chromium. Metal-induced toxicity in the lung can result in long-term, if not, permanent scarring of the lungs [13].

## Review

The ingredients of vape that are suspected of contributing to the development of vaping-related illnesses are THC and vitamin E acetate. THC is an ingredient used in many vape products, and many patients experiencing vape-related complications have admitted to using THC-containing products in the past, leading the FDA to believe that THC may play a role in the vape-related illness outbreak. The FDA has issued a public warning to stop using THC-containing vape products, as the compound may be contributing to lung illnesses related to vaping. Specifically, vitamin E acetate is most commonly used as an additive in THC-containing vape/e-cigarette products; vitamin E acetate is an oily chemical added to THC vaping liquids used to thicken or dilute them. A vape-related injury concerning a teenage boy in Canada has recently gained the media’s attention as well. The 17-year-old boy vaped “intensively,” adding THC to his devices. He initially showed symptoms aligning with bronchiolitis (lung condition normally caused by a bacterial or viral infection), but many patients that have vape-related illnesses in the United States have experienced damage to the alveoli; this type of injury was not found. Instead, his case aligned more with an injury called “popcorn lung,” an ailment most commonly seen in factory workers of microwave popcorn plants nearly 20 years ago. This new vape-related case calls for further exploration into the toxicity of vape liquid, as the patient’s condition could have been caused by the THC added to the vaping devices, or the chemical that affected factory workers in the past - diacetyl. Diacetyl is present in many e-cigarette flavors [14]. The American Lung Association has called for the FDA to require that diacetyl and other hazardous chemicals be removed from e-cigarette cartridges. 

While it is still widely debated which particular component of vape liquid is the cause of illness, vitamin E acetate, specifically, has been identified as a potential culprit in vape-related illnesses. The New York Times recently reported an analysis of lung fluid samples from 29 patients with vaping-related illnesses (including two who died), and the analysis suggests that vitamin E acetate is a "very strong culprit" in causing lung injuries. The lung fluid samples were collected from patients across the United States so that these findings may have implications nationwide. Moreover, Dr. Anne Schuchat, principal deputy director of the CDC, explained, “For the first time, we have detected a potential toxin of concern, vitamin E acetate, from biological samples from patients… The analysis provided evidence of vitamin E acetate at the primary site of injury in the lungs” [15]. Vitamin E acetate is sticky, giving it the ability to remain in the lungs. THC was also reported to be found in 82% of samples from 28 patients, which was remarkable as THC tends to leave the lungs quickly [16].

The evidence on how vitamin E acetate affects the lungs of vape users is notable because vitamin E acetate has been acknowledged as a majorly harmful chemical that may be contributing to vape-related illnesses and deaths. Lung scans have revealed different outlines of lung parenchyma suggesting possible different processes in injury. One pattern points to lipoid pneumonia which can occur with lipid containing ingredients or oils aerosolized into the airways causing inflammation and compromised function [17]. The respiratory epithelium has a complicated network of extracellular membranes essential for breathing and survival. Surfactant membranes form a stable monolayer at the air-liquid interface, reducing the surface tension at the air-liquid interface, therefore stabilizing the lung against collapse and helping lungs expand. Oil in the lung interferes with this ordered/disordered lipid phase coexistence in lung surfactant with alterations in phase coexistence [18]. The American Medical Association has made calls for a ban on vaping products, and Washington state has now banned vape products containing vitamin E acetate, thought to be linked to illness [19-20]. Although the substance is not banned in the United States and has not been officially declared as a deadly substance, many states are making advances to ban the use of the chemical in vape products. States like Massachusetts are considering a ban on flavored tobacco and vape products, and in New York, Manhattan is expected to become the largest city to ban all vaping flavors except tobacco. Other states that have already banned the use of vitamin E acetate in vape products include Colorado and Ohio. Greater public awareness of this deadly condition helps with implementing comprehensive, population-based interventions for this preventable disease.

As of November 13, 2019, there were 2,172 confirmed and probable lung injury cases "associated with the use of e-cigarette or vaping, products as reported by 49 states (all except Alaska), the District of Columbia, Puerto Rico, and the U.S. Virgin Islands as reported by the CDC. 42 deaths have been confirmed in 24 states and the District of Columbia: Alabama, California (4), Connecticut, Delaware, the District of Columbia, Florida, Georgia (3), Illinois (4), Indiana (4), Kansas (2), Massachusetts (2), Michigan, Minnesota (3), Mississippi, Missouri, Montana, Nebraska, New Jersey, New York, Oregon (2), Pennsylvania, Tennessee (2), Texas, Utah, and Virginia" [21]. Vaping, may also have harmful psychological effects with a strong association between vaping, major depression and suicidal behavior as reported in a large new study [22]. The likely contributing culprit, nicotine. Prevalence of lung disease attributable to vaping is likely under reported as cases brought to the CDC are some of the most severe. For now EVALI remains a diagnosis that is made after exclusion of other conditions and needs to be reported to the CDC.

## Conclusions

Tobacco use continues to devastate public health. Given the popularity of vaping and e-cigarettes, urgent public health call to action to this condition is needed, especially in light of the EVALI/VAPI death complications. Identification of vitamin E acetate and other possible hazardous chemical additives such as diacetyl as the contributors in the vape-related lung illness outbreak is critical to both physician and public understanding of the dangers of vaping. Identifying the particular substances in a vape that can cause lung illness and/or complications from vaping can be the crucial step toward identifying and eliminating this fatal condition, which continues to lack formal diagnostic criteria and standardized therapy. Teenagers are particularly vulnerable to the addictive flavors to nicotine. Given that many e-cig flavors, many found in candy and popular soft drinks, the addition of flavorings are likely preying on e-cigarette experimentation in young people.
